# Modelling Farm Animal Welfare

**DOI:** 10.3390/ani3020416

**Published:** 2013-05-16

**Authors:** Lisa M. Collins, Chérie E. Part

**Affiliations:** School of Biological Sciences, Queen’s University Belfast, 97 Lisburn Road, Belfast, BT9 7AL, UK; E-Mail: cpart01@qub.ac.uk

**Keywords:** simulation, risk assessment, welfare assessment, systems modelling, conceptual model, scenario modelling, optimisation, animal welfare

## Abstract

**Simple Summary:**

In this review paper we discuss the different modeling techniques that have been used in animal welfare research to date. We look at what questions they have been used to answer, the advantages and pitfalls of the methods, and how future research can best use these approaches to answer some of the most important upcoming questions in farm animal welfare.

**Abstract:**

The use of models in the life sciences has greatly expanded in scope and advanced in technique in recent decades. However, the range, type and complexity of models used in farm animal welfare is comparatively poor, despite the great scope for use of modeling in this field of research. In this paper, we review the different modeling approaches used in farm animal welfare science to date, discussing the types of questions they have been used to answer, the merits and problems associated with the method, and possible future applications of each technique. We find that the most frequently published types of model used in farm animal welfare are conceptual and assessment models; two types of model that are frequently (though not exclusively) based on expert opinion. Simulation, optimization, scenario, and systems modeling approaches are rarer in animal welfare, despite being commonly used in other related fields. Finally, common issues such as a lack of quantitative data to parameterize models, and model selection and validation are discussed throughout the review, with possible solutions and alternative approaches suggested.

## 1. Introduction

The use of models has greatly advanced in many areas of the life sciences in recent decades, but the range, complexity and type of models developed for use in animal welfare is comparatively poor. This is surprising given the similarities between the aims of animal welfare and those of, for example, the closely related field of animal health epidemiology, where there is great interest in using models (mathematical, statistical and assessment) to understand why, where, how, when and who will be affected by disease outbreaks, and to explore potential control strategies (e.g., stochastic simulation modelling [1,2,3]; decision tree modelling [4,5]; and network modelling [6,7]). In the field of animal welfare, our aims are principally to understand why, where, how, when and who will be affected by the multitude of species-specific welfare problems, and what control strategies we can put in place to prevent these problems arising. However, despite this, we do not see the same type of predictive model as is so often used in disease epidemiology being used to make predictions of welfare problems. One possible reason for this might be the relative lack of data. However, predictive disease models are often based on input data collected from largely varying sources, of varying quality and reliability and combined from multiple previously published scientific studies. 

Other related areas of academic research which frequently utilise models, are agricultural economics and sustainability. Here, we see a range of models being developed to explore, for example, the potential margins associated with different farm types and practices, often incorporating elements of animal welfare (although these are usually highly simplistic). These large, multi-factor models are again mostly developed using input data from a large range of sources, of varying quality and reliability. For example, the use of seven, predominantly resource-based, parameters to score animal welfare in a model of sustainability [8] is, perhaps, not representative of what most animal welfare academics would consider the most critical input data for a model of this type. 

Given the breadth of modelling techniques available, and the objectives these models set out to meet, it is perhaps a misnomer to talk of “models” as if they represent one approach. In other fields, such as ecology, where modelling is far more commonly used, it is recognised that models really fall into three main types: detailed (“synthetic”), “minimal” systems, and “minimal for ideas” [9]. Here, the models are defined by what they set out to do. Detailed, or “synthetic”, models aim to produce a detailed description of the sub-components that make a system and how they interact to produce the overall working system. “Minimal” models for systems aim to explain certain types of system, but tend to ignore many characteristics of the real life system and, as such, are not designed to produce specific, detailed predictions. Finally, “minimal models for ideas” are for exploring concepts without anchoring it to a particular species or type of system. Again, this type of model, which includes some of the most famous models in ecology, such as the Lotka-Volterra model, is not meant to produce testable predictions or to be applied to real life situations. We see examples of each of these types of model in the farm animal welfare literature and these will be discussed in section two. 

Modelling in farm animal welfare has mostly fallen into the frequentist statistical type, in other words, quantitative analysis of an experimentally derived, or longitudinally collected data set e.g., [10,11,12,13,14,15,16,17,18], where the main aim is to determine which abiotic and biotic factors in an animal’s environment are associated with the development of poor welfare, or change in welfare state, and which indicators can be used to determine welfare state reliably. To date, there has been little use of Bayesian approaches (though see [19,20,21]). In recent years, there has been an increasing trend to also use more statistical and engineering type methods in the formation of welfare indicators e.g., [22,23,24,25,26]. Some of these types of studies have been reviewed and discussed previously e.g., [22]. In this review article, we focus on the other types of models being produced in the field of animal welfare—namely, the assessment models, simulation, optimisation, scenario, systems and conceptual models. This paper is not intended to provide an exhaustive review of all modelling approaches. Rather, the models reviewed herein are those considered to be most promising in the field of farm animal welfare science. We will briefly describe each type of model, outline the types of questions they could be used to answer—highlighting the potential drawbacks associated with the approach—and provide examples from the scientific literature of their use in animal welfare research. Finally, we will discuss where the main gaps in farm animal welfare modelling lie, and suggest possible reasons why this may be and how we might go forward. The aim of this paper is not to provide an instruction manual on using the different modelling approaches, but to open discussion between empirical and theoretical researchers, and to forearm empirical researchers with a set of questions that they can aim to answer with theoretical approaches. 

## 2. Models in Farm Animal Welfare

### 2.1. Assessment Models

#### 2.1.1. Risk Assessment

One of the most common types of model used in animal welfare research, particularly at the science-policy interface, is risk assessment. Risk assessment characterises the probability of a negative event occurring and quantifies the consequences of such an event. Ultimately, it provides a means of comparing different welfare problems both at the level of the affected individual and at the population level, within and between species, based on a number of key factors [27,28,29,30,31]. They are therefore frequently utilised to produce lists of welfare priorities that are based on scientific research, for policy makers to consider. The European Food Safety Authority’s (EFSA) Panel on Animal Health and Welfare (AHAW) has, for instance, published risk assessments of welfare in dairy cows [32], farmed fish [27,28,33], fattening pigs [34], beef cattle and calves [35], broilers [29] and broiler breeders [30], in which aspects of housing, husbandry, management, stunning, slaughter and genetic selection, amongst other factors, were assessed. 

In risk assessment terminology, welfare problems are caused by a series of “hazards”. The fast growth rate of standard commercial breeds of broiler chicken may be considered a potential welfare hazard with possible consequences including skeletal disorders, sudden death syndrome, ascites, high body mass and muscle disorders [36]. In risk assessment, each identified hazard is characterized based on three factors: intensity of consequences, duration of effect of consequences (either as an absolute value if comparing within a breed or species, or as a proportion of lifetime if comparing between breeds or species; see [37] for an example) and prevalence (the proportion of affected individuals at any one time). Quantification (even in its simplest form, as categorical variables with groups including “mild”, “moderate”, or “severe”) allows consequences and their impact on the animals experiencing them to be compared. However, in this basic form of risk assessment, specific details of the hazards are not considered. A more accurate risk estimate can be obtained by also including information about the hazard itself in the calculation, such as estimates for the duration and probability of exposure to the hazard. In providing a quantitative, or even qualitative, value for each of these factors, the aim is to produce an objective estimate of risk for a series of potential welfare hazards.

However, as outlined in Collins [38], there are currently three main issues with welfare risk assessment procedures: (1) An incorrect assumption of independence between one hazard and its consequences and another hazard and its consequences. Although it is almost certainly true that some combinations of hazards will be truly independent, in most cases there is at least some degree of non-independence between hazards and, indeed, some overlap in the types of consequences emerging from different hazards. The effects of non-independence on a risk assessment procedure are a risk score that is either over- or under-estimated, typically as a result of conflated prevalence information, but also, and rather less easily measurable, conflated intensity information. (2) The common use of expert opinion to quantify each of the hazard characteristics, in lieu of purely data-based estimates, is a potential source of bias and unreliability in the risk assessment process. Of course, data-based estimates may also be subject to bias arising from selection, collection, analysis and/or interpretation of data [39]. However, just as expert opinion is gathered from numerous individuals or groups, ranging in field of expertise, data-based estimates should be based on a range of studies and datasets derived from numerous sources. Doing so would reduce the impact of isolated instances of bias. Collating scientific evidence by means of systematic reviews would involve quality assessing studies whereby those at risk of bias would be excluded, and meta-analysis enables the potential effects of biases to be formally examined [40]. (3) Level of uncertainty and variability are frequently not calculated in welfare risk assessments. This makes assessing the information they contain very difficult. Did all experts agree 100% on all the scores for the hazards, or was there a large divide in opinion? Without this information, it is very difficult to assess how confident one can feel in the result. This information would routinely be given in a typical statistical analysis, in the form of standard errors, confidence intervals and sample sizes. The same should be true for risk assessment procedures. However, above all, it is particularly true when quantifying intensity of suffering. This is essentially because the strength and reliability of expert opinion is dependent on the scientific research that has been published on a subject. There is no single welfare indicator that can reliably measure welfare across different contexts and across different species. Instead, we are reliant on multiple indicators that can often give conflicting results regarding affective state. Thus a random group of experts would be expected to differ in their opinions on intensity more than they might on something more easily quantifiable, such as prevalence.

To summarise, at this stage, animal welfare risk assessment is often used as a conceptual modelling tool to allow very different welfare problems to be compared. However, it could be made more quantitative and less subjective using systematic searches and data mining tools, to extract information from the resulting large volume of peer- and non-peer reviewed papers, and meta-analysis of the extracted data. 

#### 2.1.2. Welfare Assessment

Welfare assessment is typically the term given to the *in situ* appraisal of an animal’s affective and physical states, or most commonly, to the group assessment of welfare at the herd or flock level, e.g., [41] or at the system level e.g., [42]. Welfare assessment systems can be categorised on the type of information they collect: (i) resource-based; and (ii) animal-based assessment systems. Resource-based systems are primarily interested in the inputs provided for the animals [43]. Input factors can often be assessed with a high degree of reliability [43], but are perhaps more suited to informing farmers about possible prevention and solution strategies to problems, rather than identifying and assessing the health and mental well-being of the animals [44]. It has been shown that resource-based parameters alone are not sufficient to assess welfare e.g., [45] and can be thought of as providing a “risk assessment” (or “housing condition assessment”) as opposed to a welfare assessment [46,47,48]. This could, however, be overcome by using resource-based parameters that have strong links with animal-based measures and can reliably predict welfare at the individual level [49]. Animal-based measures of physical condition and health, disease status, and behaviour e.g., [44,49,50,51,52,53] are also limited when used alone as they cannot conclusively identify the causes of poor welfare [49].

Assessment models have been developed for cattle e.g., [41,46,47,51], pigs e.g., [42,52], poultry e.g., [48,53,54], farmed mink and foxes [55], and to evaluate enrichment materials for pigs (RICHPIG e.g., [56]). Other researchers have modified existing models for use in their studies. For example, Munsterhjelm *et al.* [57] modified the ANI-35-L for use in commercial pig production systems in Finland, and Stott *et al.* [58] modified the Welfare Quality^®^ criteria for use in their study on extensive sheep farms. 

Some models focus on resource-based indicators (e.g., ANI-35-L [59]; FOWEL [54]; SOWEL [42], but see Bracke [60]), others on animal-based measures (e.g., Welfare Quality^®^ project models [61]) and others on both direct and indirect welfare indicators e.g., [46,49]. Aerts *et al.* [62] proposed a more “holistic” welfare assessment framework where the housing system (environment), the stockholder, and “the whole animal” are assessed; the former through the ANI-35-L [59] and the latter through Free Choice Profiling (FCP [63,64]). The proposed model does not focus on ranking attributes or on the overall welfare score but, rather, on how welfare can be practically improved at the farm level [62]. While clearly advantageous, this focus, together with the qualitative “whole animal” assessment (FCP [64]), might render the model unsuitable for categorising farms for food labelling systems and welfare certification schemes. 

Both the quality of stockmanship and human-animal interactions influence animal welfare and are, therefore, important parts of an overall welfare assessment [47,49,59,65]. However, obtaining direct and objective measures on-farm may be difficult [47,48,59]. Stockmanship has previously been measured indirectly through an assessment of the animals’ or the environment’s cleanliness or condition; for example, scoring plumage or hoof condition, cleanliness of feeders, *etc.*, e.g., [47,48]. The Welfare Quality^®^ models generally assess the human-animal relationship (HAR) through animals’ “fear of humans” (*i.e.*, the assessor) on-farm (e.g., using the avoidance distance test in broilers and laying hens) [51,52,53]. Although an informative measure [66] that quantifies a qualitative phenomenon, direct observation of farmer-animal interactions (potentially scoring aspects of the farmers’ animal handling as in Welfare Quality^®^’s [51] scoring of “coercion”, *i.e.*, use of electric goad, stick, *etc.*, in the handling of cattle at slaughter) may also be required to help identify the specific causes of animals’ fear of the assessor and to provide specific advice to farmers that might improve the HAR. Another approach to incorporate human-animal interactions was developed by Stott *et al.* [58], who applied an adapted Service Quality Model (SQM), which is typically used in management science, to assess the interaction between profit and welfare on extensively housed sheep farms. The SQM considered the impact of farm management on sheep welfare, including stockperson “empathy” and “knowledge and experience” [58]; factors which are rarely included in welfare assessment models. The SQM focused on the gap between expectations and observed performance, where animal welfare was considered to be a function of quality of service provided [58]. The results from this were then compared with results using an adaptation of Keeling and Veissier’s [67] Welfare Quality^®^ criteria (Qualitative Welfare Assessment, QWA). However, although the two methods were reported to give “complementary results”, this does not appear to have been explicitly tested. 

Some, if not most, welfare assessment models provide an overall welfare score (e.g., the Integrated Diagnostic System Welfare model [46]; SOWEL e.g., [42]), which involves weighting the multiple input factors. Weighting can be based on expert opinion, but this may introduce bias [68] and may not reflect the views of other stakeholders [69]. Drawing experts from a range of different fields is important in order to balance viewpoints [70]. However, if experts are brought together to choose and/or weight criteria, the group’s consensus view may actually be the view of a persuasive minority [71]. On the other hand, there can be considerable variation in the weightings applied by experts [72] calling into question the value of this approach. Measuring and reporting variation in opinion, alongside experts’ confidence in their weighting of assessment criteria, as in Bracke *et al.* [72], would enable potential users to assess the validity of a model based on expert (or other) opinion. Conjoint analysis, frequently used in market research [73], has been used to weight welfare parameters based on opinion [74,75]. Here, participants evaluate “welfare profiles” rather than rank or weight individual attributes, which more closely resembles “real world” decision-making [75].

One approach to objective weighting of criteria is semantic modelling (SM), which was used in the development of the SOWEL [42], FOWEL [54] and RICHPIG models [56] and to assess the importance of wallowing for pig welfare e.g., [76]. Bracke *et al.* [77] also demonstrated how animal welfare risk assessment might benefit from employing SM methodology. In SM, assessment criteria weightings are based on “scientific statements” (*i.e.*, statements extracted from the literature denoting empirical observations of some aspect of welfare under particular conditions) e.g., [42,54] and, in later models, include a measure of uncertainty (*i.e.*, strength of each statement) [56]. The validity of the resulting model can then be tested through, for example, comparison with expert opinion e.g., [72,78,79], experiments designed to determine the importance of assessment criteria from the animals’ perspective e.g., [80], and sensitivity analysis [56]. 

It has been suggested that welfare assessment models and their individual criteria should be treated as diagnostic tests and evaluated in terms of their sensitivity and specificity in identifying an animal’s welfare status, as employed by Nyman *et al.* [41]. Of course, unlike diagnostic tests for the presence of particular pathogens (such as bovine tuberculosis testing in cattle), welfare assessment models are not testing an individual for the presence or absence of a single parasitic entity existing within, or on, their being. Instead, it may be more accurate to consider welfare assessment models as being akin to diagnostic tests for non-parasitic conditions, such as certain mental health disorders in humans, which require triangulation of clinical signs and symptoms to reach a diagnosis [81,82,83]. The optimal, validated assessment model would include the most sensitive and specific criteria, with established cut-off points for “good” and “poor” welfare [41]. It would be important for the model to highlight those animals close to a cut-off point, so that they can be closely monitored. Furthermore, as the good welfare of one animal cannot ethically offset the poor welfare of another within a herd (at least from a deontological viewpoint), a measure of variation is necessary when scoring welfare at the farm level [70]. An alternative approach is to base the assessment on the lowest 25% of welfare scores within a herd [47,48], although this then gives little indication of overall herd welfare. For example, when assessing welfare problems such as lameness, mastitis or foot rot, where prevalence within a herd can be high, the bottom 25% (and more) could all be considered to have compromised welfare. 

Although a range of welfare assessment models have been developed, their widespread use in other areas of research is not apparent. Animal welfare is considered an important factor when assessing the social sustainability of farming systems [84]. However, the assessment of animal welfare within agricultural sustainability research varies widely from the use of a limited few measures e.g., [85] to the use of a developed model supplemented by additional criteria [86] to behavioural observations and physiological indicators [87]. Clearly, there is need for multidisciplinary work in this area, and for increased collaboration between farm animal welfare scientists, economists and researchers in sustainability and food security.

### 2.2. Simulation Modelling

The term simulation modelling is extremely broad and, in actuality, could refer to many of the kinds of model that will be described in the following sub-sections. However, in the more selective sense, a simulation model is one that seeks to recreate patterns observed in real-life with the input of selected, often simplified, variables that are thought to be responsible (at least in part) for the production of the resultant pattern. These models are typically described as “stochastic”, for models that include one or more parameters with random values drawn from identified probability distributions; or “deterministic”, for models that include no random variables and no randomness. Simulation models can also be described as “dynamic”, if they include time as a variable, or “static” if time is not included. Dynamic models are typically represented with differential or partial differential equations. Further to this, dynamic models can be described as “continuous” or “discrete”, depending on whether changes in time are represented with a continuous interval, or discrete time steps (events), respectively [88,89]. Models may further be described as “top-down”, where the developers start with a general overview of a system (the “big picture”), but do not have details of the component subsystems. This type of model tends to be formulated with a large number of differential equations. By comparison, many modern models are “bottom-up”, such as those used in agent-based modelling (ABM) where individual units (e.g., animals, though one could potentially also consider pens, houses or farms as separate units) are programmed to behave according to a set of probability-based rules and can interact with other units (though ABM can also be data-based [90] or use deterministic rules [91]). The resulting emergent patterns at the global level of the model can then be compared with patterns observed in real life. Bottom-up, generative models such as ABMs have been used in many different fields, including disease dynamics e.g., [92,93], evolutionary and social processes e.g., [94] and to describe financial markets e.g., [95]. 

In farm animal welfare, simulation modelling has been used to estimate the costs of welfare improvements for commercial pigs [75] and the economic and welfare impacts of foot disorders [96,97] and foot disorder interventions [98] in dairy cattle. Den Ouden *et al.*’s [75] model simulated all stages of the production chain (farrowing, fattening and slaughtering) as well as transportation between stages and time spent in lairage. However, effective models need not be complicated. Waterhouse [99] presented a simple model to estimate the time and effort required to supervise ewes during lambing, and the provision of this care, at different stocking densities. Febrer *et al.* [100] simulated social attraction, aversion and indifference in broiler chickens housed at different stocking densities, and compared these simulations to observed spatial distributions of real chickens, to investigate if broilers prefer personal space or the closeness of other birds. Likewise, and as with the Febrer *et al.* [100] model, based on just two parameters (in this case, numbers of birds at the trough currently and 5 seconds previously), Collins and Sumpter [101] showed that broiler chickens demonstrate a Mexican wave-like property in their feeding dynamics around a trough at different stocking densities. Both models [100,101] suggested that social dynamics and clustering occur independently of stocking density. 

The development of more complex models may be somewhat constrained by a lack of available quantitative data, which, for example, prohibited the modelling of some intervention measures and interactions between foot disorders in Bruijis *et al.*’s [98] study. However, gaps in the literature are to be expected, and existing models can be updated as scientific research progresses. At the same time, we can make good use of existing scientific knowledge by conducting meta-analyses to produce parameter estimates—this could prove particularly effective for studies aiming to investigate the welfare effects of different environmental conditions, or in different breeds, for example, which have not been explicitly compared in a single experiment, but have been partially investigated across several studies. 

Modelling the impact of various measures on animal welfare can be difficult due to the range of factors that can interact to influence welfare and the difficulties in estimating their effects [98]. Farmer attitude and knowledge, for example, are complex factors to model [98]. Furthermore, some types of welfare issue may be more straightforward to simulate than others, particularly if they can be reliably quantified. For example, those relating to physical health and disease, where clinical signs can be observed (or tested for) in a group with known estimates of specificity and sensitivity, or behavioural indicators that are validated and quantifiable, such as performance of abnormal behaviours, or social dynamics and clustering. 

Selecting and validating the best simulation model can sometimes prove difficult. In an ideal world, all simulations would be tested using sensitivity analysis, and validated against an independent dataset after development. In practice, this is often not performed prior to publication if the model is based on the only available, existing dataset for a particular problem at that time. However, even if this is the case, statistical methods such as cross-validation, sub-sampling and bootstrapping could be utilised to create alternative datasets for model validation [102,103,104]. Of course, a simulation is highly unlikely to make predictions with 100% accuracy. Where agreement between the model output and a dataset is relatively mediocre (measured with goodness-of-fit), one could draw conclusions from the differences between the conditions in the model and the dataset, rather than from model output *per se* [105]. An alternative approach may be to improve goodness-of-fit. Goodness-of-fit is often improved by including additional parameters in a simulation, but this can often make interpreting the model output difficult [106] or lead to overfitting [107]. Furthermore, goodness-of-fit doesn’t tell us if the model itself is any good—it only tells us how well (or rather, how badly) the predicted values match those in an independent dataset, and overall fit can be good even if there are some areas where the fit is very bad [108]. 

There is great potential for simulation modelling to be used to a much wider extent in animal welfare research. For example, given the expected depletion in oil reserves [109,110,111,112], changes in fossil fuel [111,113] and electricity prices [114], projected increases in demand for meat [115] and the potential effects of changes in climate [115,116,117,118] over coming decades, a long-term evaluation of the possible effects of these factors on farm animal welfare could prove extremely valuable for welfare scientists, policy makers and industry, particularly if potential intervention or amelioration strategies could also be investigated. 

### 2.3. Optimisation Models

As the name suggests, optimisation models are developed to find the optimal solution to a defined problem and typically take the form of linear and dynamic programming in livestock science [89]. Linear programming (LP) models reveal the optimal set of variables that maximises (or minimises) a particular function under specified constraints [58,119]. Dynamic programming (DP) models are also based on mathematical optimisation, where a larger problem can be broken down into multiple smaller subproblems, which can in turn be broken down in a recursive manner. Two classic examples include Dijkstra’s shortest route algorithm [120], and the Tower of Hanoi recursive solution. Farm animal welfare research employing optimisation models have principally focussed on the economics of animal welfare. Unlike simulation models, which can be used to simulate the behaviour of a system, animal or disorder and estimate the impacts of manipulating various input factors, optimisation models are designed to solve a specific problem, optimising a particular function and identifying the best possible strategy or outcome [89]. 

Langford and Stott [121] used DP to maximise dairy farmers’ economic gain through determining the optimum (financial) decision to keep or replace a heifer at each parity over a 20-year cycle. Modelling different farm scenarios (high and low rates of infertility, mastitis and lameness) allowed estimates of the long-term effects of improving cow welfare. Several other studies have also used these models to maximise financial return at the production level. For example, LP models have been used to minimise the costs of improving pig welfare [122], to determine the most profitable body condition at which to maintain sheep [123] and to estimate the maximum profit potential of individual sheep farms [58]. Vosough Ahmadi *et al.* [124] used LP to investigate trade-offs between economics (profit) and sow welfare under different farrowing systems, and to provide a framework for designing economically feasible, high welfare systems. Other studies have used LP to estimate the price of pork produced under high welfare farrowing systems [125], to compare dairy farming systems and to estimate the impact of management changes on economics and animal welfare, amongst other factors [126]. 

There appears to be great potential for the use of optimisation models in future research, where animal welfare science and economics combine to find win-win scenarios for farmer and animal [124]. However, future LP models might also be used to examine potential trade-offs between animal welfare and environmental impacts e.g., [127] and aim, for example, to minimise GHG emissions under animal welfare constraints. One of the main difficulties with developing such optimisation models, as with all other models discussed in this paper, is in pinpointing the welfare components for inclusion and in determining the complex effects that different changes would have on the animals’ well-being [69]. Changes perceived to be beneficial by the public, like banning gestation crates for sows and battery cages for laying hens may, in practice, improve some aspects of the animals’ welfare and reduce other aspects [69,128], which further emphasises the need to include multiple animal-based measures in models of animal welfare and, where possible, to base each relationship on scientific evidence rather than on opinion. 

### 2.4. Scenario Modelling

Scenario modelling is not really a separate type of modelling used in farm animal welfare research, so much as a method that often overlaps with several of the approaches already discussed (simulations, optimisation models). It may offer an alternative approach to forecasting whereby, rather than trying to predict future events, it compares a variety of alternative futures (or potential solutions), asking the “what if…?” question [129]. The effects of different scenarios on the model outcomes are estimated and then compared with the basic simulation (often reflecting the current situation). For example, in one study, three alternative future scenarios for organic dairy farming in Denmark (focussing on profit, animal welfare, and environment) were modelled and evaluated in terms of their economic and environmental impacts. Model simulations and expert knowledge were used to parameterise the scenarios [127]. Alternative cow welfare and pig performance scenarios have been investigated, using DP and LP, to estimate the effect on farmer income [121] and on the price of pork produced under different farrowing systems [125], respectively. 

Although farm income has risen over recent years, farming is still not a highly profitable business [130]. Therefore, the balance of economic viability and good animal welfare is a tight line to tread. Scenario modelling provides a means of comparing alternative solutions (e.g., increased retail price of animal products, additional Common Agricultural Policy (CAP) Single Farm Payments, introduction of a national tax, complete shift to large-scale farming) in terms of their impacts on the utility (*i.e.*, well-being) of all stakeholders (*i.e.*, animals, farmers, retailers, consumers, citizens) and on the national economy. Scenario modelling might also be used to compare alterative future solutions that would enable the livestock industry—particularly in countries with already highly-intensive production and low welfare standards (e.g., China [131])—to meet the increasing demand for animal products [115].

Comparing multiple different possible futures may, however, be hampered by the fact that scenario modelling does not incorporate any element which could determine which of the futures is most likely, or optimal. Rather, it simply outlines what the different possible conditions could be under each of the different scenarios. It is then left open to interpretation which of the possibilities would be most ideal or most probable under current circumstances. This facet means that scenario modelling is an excellent choice for making value-free judgements within the model construct, although it also means that judgements must be made post-hoc, perhaps rather more subjectively than would be the case with other modelling methods.

### 2.5. Systems Modelling

Systems modelling is the analysis of complex systems, and investigation of how functionally different sub-processes within a system integrate and interact to produce a coherent system [132,133]. Unlike most other types of modelling discussed in this paper, systems modelling is almost by definition interdisciplinary, and is based on the underlying principle that to fully understand a system, one must understand it at different organisational levels [132] such as at the molecular, cellular, organismal and species level, all within one model. To date, the majority of systems biology studies have focussed on, for example, drug discovery [134], forecasting and diagnostics in plant, animal and human disease [135], and the design of bio-products such as bio-fuels [136]. 

This approach has the potential to make major contributions towards the development of more sustainable farming systems [137]. For example, the Sustainable and Integrated Management Systems for Dairy Production (SIMS_DAIRY_) model brings together existing models, equations and “score matrices” that reflect economic, environmental, ecological and social factors relevant to farm sustainability (including animal welfare), involving simulation, optimisation and scenario modelling at the farm level [138,139]. The SIMS_DAIRY_ model has been used to compare the potential of improving nutrient management with plant and animal genetics in enhancing the overall sustainability of UK dairy farms [139]. In another study, the SIMS_DAIRY_ model was used to compare three simulated organic systems with two intensive systems (differing in their use of nitrogen fixing plants and use of fertilizers and pesticides) and compared, for example, the estimated GHG emissions, farm income, biodiversity and animal welfare of simulated farms [8]. Neither of these two studies focussed on animal welfare, but the model incorporates an animal welfare score matrix [139]. The factors assessed to calculate animal welfare were mainly resource-based. There is clearly much scope for further development of this sub-model [8], perhaps by integrating an animal-based welfare assessment model (e.g., the Welfare Quality^®^ model for cattle [51]). 

Systems modelling facilitates our understanding of how different parts of that system interact and enables a combination of changes, within different parts of the system, to be evaluated [139]. Developing systems models can be very time consuming and the end-product is highly specific. Nonetheless, adopting a systems (or, perhaps, a network) approach could provide valuable tools to improve our understanding of how animal welfare interacts with other measures of sustainability and, particularly: (1) to estimate how specific improvements in farm animal welfare would impact on the environmental, ecological and societal sustainability of the farm; (2) to estimate how improvements in the economic, environmental, ecological and/or societal sustainability of the system would impact on animal welfare; (3) to identify specific improvements in animal welfare that, if implemented, would improve overall sustainability of the farming system (leading to win-win scenarios that are more likely to be adopted); and (4) to identify combinations of factors within different parts of the system that could be changed to optimise animal welfare and overall sustainability [139]. 

### 2.6. Conceptual Models

Conceptual models allow consideration of the fundamental activities of a system without being tied to details of the physical reality of that system. Checkland [140] defined a conceptual model to be “a statement of what is logically and necessarily implied by the [root] definition. It is not a recommendation of what ought to exist nor of what does exist in the real situation.” Thus, economic models exemplify this type of approach. Conceptual models identified in the farm animal welfare scientific literature are diverse in subject matter, including human and animal willingness-to-pay (WTP) for improved animal welfare [141], motivation for sucking behaviour in calves [142], stereotypy development and maintenance under feed restriction in pigs [143], a quantitative genetic model of animal learning [144] and a model to aid artificial selection for enhanced welfare in pigs [145]. Other conceptual modelling studies have considered the impacts of farmers’ perceptions, attitudes and behaviour on their choice of whether to implement welfare improvements [146], a matter which has generally been overlooked in other models of animal welfare. Other researchers have applied a conceptual socio-psychological model (the “theory of planned behaviour”) to understand farmers’ decisions, and underlying motivations, to change husbandry practices, such as group housing for pregnant sows [147], alternatives to mulesing in sheep [148], and to identify interventions that might encourage farmers to implement such changes [147].

Lusk [149] conceptualised the construction of a distinct market for animal welfare, separate from the trade of meat products (*i.e.*, the development and trade of animal well-being units [AWBU]). Simple algorithms to calculate AWBU on a farm were proposed, building on existing welfare assessment models. To establish a successful market, clearly AWBU need to reflect consumer preferences [149] and this is where an assessment model of farm animal welfare based on public perceptions e.g., [150] could be very useful. This model may seem idealistic, and it’s realisation improbable. This is recognised by Lusk [149] who exemplifies the existence of such a market. While it is unlikely that this idea will overtake the drive for welfare labelling of animal products, it gives us food for thought on additional ways in which animal welfare improvements could be realised in the future. 

A full discussion on economic models and their contribution to farm animal welfare research is beyond the scope of this paper, but would be extremely worthwhile. However, McInerney’s [151] model is noteworthy here. This conceptual model illustrates a theoretical relationship and, specifically, conflict between farm animal welfare and productivity (*i.e.*, animal *vs.* human benefit). Here, improvements in productivity and animal welfare go hand-in-hand up to a certain point, after which further increases in productivity will reduce the welfare of livestock. The question is; where, and how, do we strike the right balance between the well-being of humans and that of the animals we eat [151]? There is great potential for mathematical modelling to help us answer this question, drawing on both economics and animal welfare science [124]. Such work has already begun with a number of models outlined above (e.g., the optimisation models developed by Langford & Stott [121], Kingwell [123] and Vosough Ahmadi *et al.* [124]), which identified win-win scenarios for farmers and livestock at farm, or system, level. Such interdisciplinary, bio-economic models have the potential to make important and practical contributions towards improving farm animal welfare.

Thus, conceptual models can provide a theoretical basis to help guide future research, propose potential solutions, collate empirical evidence and illustrate ideas of how different factors might interact. There is, therefore, scope to develop conceptual models within all areas of animal welfare research. However, unlike the other models discussed in this review, conceptual models, not being rooted in physical reality, can often not be explicitly tested. 

## 3. Discussion

The aim of this paper was to review the use of non-statistical models in farm animal welfare research. Most of the research to date has described the development, or use, of conceptual, risk and welfare assessment models. The use of mathematical models has been limited, despite their potential to assist us in: (1) predicting when and where welfare problems are likely to arise and who they are most likely to affect; (2) determining how different components of the farming system interact to influence welfare; (3) identifying the best control strategies that we can apply to prevent welfare problems from developing; and (4) bringing together large bodies of evidence from different fields to establish links between animal welfare, economics, environmental and social sustainability in livestock farming. Here, mathematical modelling could assist us in identifying, and tackling, any existing or impending conflicts between, for example, farm animal welfare, farmer livelihood, future food security and environmental legislation.

In a review of the literature, de Boer *et al.* [152] identified some potential effects of greenhouse gas (GHG) emission mitigation strategies on animal welfare, human health, emissions, land use and other sustainability factors; but highlighted that all is far from clear. They called for an amalgamation of life cycle (sustainability) assessment (LCA) and simulation models that exist across disciplines and that reflect different levels of the farming system to fully comprehend the consequences of instigating GHG mitigation strategies [152]. The result would be akin to a complex systems model, in which component interactions (*i.e.*, cause-effect) would be modelled throughout the entire production chain at farm, crop and animal levels [152]. Such a model could prove influential in policy makers’ decisions. To ensure that animal welfare science can contribute to its development and that the animal welfare impacts of GHG mitigation are more fully understood, we need to use our existing knowledge to model the links between farm animal welfare and other aspects of sustainability, following on from the models that already exist in animal welfare literature. In doing so, gaps in knowledge will be highlighted; directing future research in this area.

Although numerous studies reviewed herein examined the economics of farm animal welfare, few incorporated consumer willingness-to-pay (WTP) for higher welfare standards as a factor in their models, which would be required for a complete economic evaluation of welfare improvements [75,122]. This may be because of the difficulties incurred in accurately estimating WTP e.g., [153,154], or due to a lack of available data e.g., [75]. However, research into consumer WTP for welfare improvements is increasing e.g., [155,156,157,158]. The values estimated in these studies could be used in future models, and to refine existing models, to identify potential win-win scenarios for farmer and animal. Given that it is ultimately the farmer’s decision whether or not to improve the welfare of animals above that required by legislation, it may also be in our interests to better understand farmers’ decision-making processes, and how the major supermarkets influence these. Here, agent-based modelling (previously used in agricultural e.g., [159] and consumer behaviour studies e.g., [160]) may be useful [161]. 

Insufficient quantitative data can cause difficulties when developing valid mathematical models [1] as the accuracy of model output depends largely on the reliability and validity of input data [162]. Input data can be collected or estimated through direct measurement, literature reviews and expert opinion. While considered the “holy grail” in medicine, meta-analysis is rarely employed in animal welfare science but, where possible, could provide more reliable estimates of input data, and of relationships between parameters, than direct measurement in a single, all-encompassing, study. As randomly controlled trials tend not to be used in animal welfare research, however, differences between studies (housing conditions, breeds, management, *etc.*) and study limitations must be identified and taken into consideration in the analysis. 

Thus, there is no need to wait for the collection of “perfect” data sets to build or parameterise models, which may be one of the reasons behind the limited use of mathematical models in farm animal welfare research to date. There are, already, many scientific papers reporting experimental data on which data mining and meta-analysis techniques could be used to parameterise a model (here, we are referring to all models, including risk assessment). Over 40 years of research has led to a wealth of experimental findings collected under various housing conditions and farming systems, with a range of species and breeds, using various welfare measures and reporting different welfare statuses. Indeed, it would be a shame not to make the most of this data. 

It must be noted that modelling is not a precise science and, in some ways, is quite subjective. Parameterising the model with “real” (observed) data will increase objectivity; however, it will also increase the risk of error in model output, and reduce its applicability to other datasets, if the dataset used to parameterise the model is not valid, or contains erroneous values that aren’t typically observed [163]. Increasing model complexity by increasing the number of parameters may result in overfitting, which can reduce the model’s predictive value [107]. Thus, there is a trade-off between bias and variance, whereby bias decreases, and variance increases, as parameter numbers grow [164]. 

It could also be said that there is a trade-off between precision and manageability [89], which will certainly apply to the dataset used to parameterise the model. However, as shown by Mackay and Lee [107], simpler models can also have the best predictive value when tested against other datasets. 

Model validation is vital, otherwise, the model is an untested hypothesis. Validating the model using an independent dataset (*i.e.*, an entirely new dataset to the one used for parameterisation) is generally considered the best approach [163]. However, this is often not possible if all available data is required to build the model. In this case, techniques such as cross-validation, bootstrapping and sub-sampling can be employed [102,103,104].

Assumptions are generally used to simplify the analyses [165] and are typically based on real data or expert opinion e.g., [166]. It is important to consider the evidence behind model assumptions when interpreting model output and drawing conclusions e.g., [166]. Models that measure animal welfare using resource-based parameters are based on the assumption that welfare can be improved through changes to the animals’ environment, or other resources bestowed on them. While this is likely true, we must also be careful not to fall into the “anthropomorphic trap”. For example, while high stocking density is considered a risk to welfare in broiler chickens [29], research suggests stocking density *per se* is generally of less importance than housing management [167]. This illustrates the necessity to base model assumptions on scientific evidence (*i.e.*, establish links between resource- and animal-based parameters [49]) and to incorporate other factors that might interact with environmental parameters to impact on the animal’s welfare, such as quality of stockmanship and the human-animal relationship [47,49,59,65]. However, where such evidence-based assumptions cannot be made, this should not discourage model development as the model can be strengthened as more data becomes available. In such cases, theoretical concepts can be combined with empirical data to build a model, and the concepts can then be used to guide future data collection to support or refine those assumptions.

We should be working towards determining the sensitivity and specificity of individual welfare indicators and whole assessment models, and establishing non-arbitrary cut-off points for good/poor welfare. Alternatively, we could adopt and adapt methods used in formulating diagnoses in human psychiatry e.g., [81] whereby criteria are grouped and individuals must meet a certain number of these criteria to be diagnosed with a particular condition. In animal welfare, this could take the form of, for example, individuals being scored against sets of criteria for good welfare, compromised welfare and severely compromised welfare (e.g., severe feed restriction [168], mobility score 3 (*i.e.*, lame) [169]). 

## 4. Conclusions

Modelling within animal welfare research has been largely focussed on the development and use of conceptual and assessment models. While there is great scope for progressing animal welfare science through integrating our existing knowledge in the development of mathematical models, we found that the use of such techniques has been limited to date. The development of “whole systems” models will require inter-disciplinary collaborations with systems biologists, economists, and sustainability and food security experts. In return, these large-scale models may have scope to influence decision-makers and, certainly, to improve our understanding of how, and where, animal welfare improvements fit into the wider context of sustainability and food security. Preliminary work towards the development of such complex models has already begun with the more specific simulation, optimisation, scenario and assessment models that have been outlined in this paper. 
